# Gender-Driven English Speech Emotion Recognition with Genetic Algorithm

**DOI:** 10.3390/biomimetics9060360

**Published:** 2024-06-14

**Authors:** Liya Yue, Pei Hu, Jiulong Zhu

**Affiliations:** 1 Fanli Business School, Nanyang Institute of Technology, Nanyang 473004, China; 2 School of Computer and Software, Nanyang Institute of Technology, Nanyang 473004, China

**Keywords:** speech emotion recognition, genetic algorithm, high-dimensional, feature selection

## Abstract

Speech emotion recognition based on gender holds great importance for achieving more accurate, personalized, and empathetic interactions in technology, healthcare, psychology, and social sciences. In this paper, we present a novel gender–emotion model. First, gender and emotion features were extracted from voice signals to lay the foundation for our recognition model. Second, a genetic algorithm (GA) processed high-dimensional features, and the Fisher score was used for evaluation. Third, features were ranked by their importance, and the GA was improved through novel crossover and mutation methods based on feature importance, to improve the recognition accuracy. Finally, the proposed algorithm was compared with state-of-the-art algorithms on four common English datasets using support vector machines (SVM), and it demonstrated superior performance in accuracy, precision, recall, F1-score, the number of selected features, and running time. The proposed algorithm faced challenges in distinguishing between neutral, sad, and fearful emotions, due to subtle vocal differences, overlapping pitch and tone variability, and similar prosodic features. Notably, the primary features for gender-based differentiation mainly involved mel frequency cepstral coefficients (MFCC) and log MFCC.

## 1. Introduction

Emotions are considered an integral and important part of human life, and they are a way to express one’s opinions and inform others about one’s physical and mental health [1]. Speech signals have emerged as a valuable source of information about a speaker’s emotions, and they have the advantage of easy recording compared to other physiological signals that require special equipment [2]. As a result, speech emotion recognition (SER) has received increasing attention.

SER systems aim to discern the potential emotional states of speakers from speech signals [3]. These systems find applications in various fields, from human–computer interaction to automated supervision and control of safety systems [4]. For instance, in remote call centers, they can automatically detect negative or positive customer experiences, and facilitate the evaluation of company services or employee attitudes. In crime investigation, they can be utilized to determine the psychological state of suspects and judge their truthfulness. In vehicles, drivers’ emotional information is extracted to improve safety. Additionally, identifying the emotional states of students in academic environments can assist teachers or intelligent virtual agents in providing appropriate responses, thereby improving the quality of education.

SER is essential for improving device intelligence, promoting personalized voice services, and achieving natural and harmonious human–computer interactions [5]. It is difficult to accurately predict, due to the complex nature of human emotions [6]. Gender is a significant contributor, because of differences in acoustic characteristics. The pitch distinction between males and females is substantial, while variations within the same gender are relatively minor. For example, it can be difficult to distinguish between a happy male voice and a calm female voice. Researchers employ features from gender and acoustics to enhance accuracy; however, this can be subject to the curse of dimensionality.

Feature selection is a common method for recognizing emotions and reducing dimensionality [7,8,9]. Metaheuristic algorithms have universal and diverse heuristic strategies [10,11], and they are powerful tools for handling complex optimization problems such as feature selection [12,13,14]. A genetic algorithm (GA) mimics the process of natural selection, in which promising individuals are selected for producing the next generation [15,16]. Binary-coded GA can be directly used to solve the selection/non-selection of features, without the need for position transformation [17,18]. Traditional feature selection methods based on GA often employ unguided crossover and mutation operators for random evolution. Unfortunately, these methods tend to generate numerous suboptimal solutions through computationally expensive fitness functions. Zhou and Hua introduced a new feature selection algorithm based on a correlation-guided GA [19]. It examined potential solutions to reduce the chance of producing inferior offspring, while classifiers validated more promising solutions to improve the efficiency of the GA. Theoretical proofs supported the claim that this method converged to the global optimal solution under very weak conditions. GAs face the issue of falling into local optima when solving high-dimensional, multi-extreme constrained optimization problems. Song et al. suggested a real-coded GA [20]. First, the sorting group selection, a simple and easily implementable approach, was presented. Second, a combinatorial crossover operator was proposed to advance the exploration of the GA, which involved a heuristic normal distribution using directional crossover, optimal individuals, and sine–cosine crossover. Third, eliminating similarities between different variables in the same dimension helped to avoid premature convergence and maintain diversity. Fourth, a mutation operator was introduced to balance global and local searches. Li and Li introduced a hybrid GA to facilitate escaping from local optima [21]. The species diversity of the initial population was computed using parallel GA and information entropy. Additionally, through parallelization, they implemented a complete information game operation to optimize the overall performance of the entire population based on information entropy and the fitness values of each subpopulation. Yan et al. considered the diversity of population fitness, crossover probability, and mutation probability in a nonlinear adaptive GA [22]. To enhance the optimization efficiency, the introduced selection operator was combined with the best–worst preservation strategies, and a method of retaining parents was proposed, to maintain the same population size during genetic manipulation. Compared to the classic GA, the proposed algorithm was more effective at eliminating extreme values and achieving faster convergence. A GA comprises selection, crossover, and mutation, and they produce a better chromosome. Mutation is one of the three main operators that is crucial for achieving optimal solutions, and it is utilized to preserve population diversity. In [23], a new type of mutation, called Zigzag mutation, was proposed to improve a GA. The Zigzag pattern indicated that the new mutation produced significant and sudden mutations in genes compared to existing mutations, and it improved the local search efficiency. Refs. [24,25] utilized a Markov chain to theoretically prove the convergence of GAs. The strong Markov property of the population sequence was derived using a mathematical model [26]. Under the condition that time approaches infinity, the convergence of the GA was proven, and its convergence rate was estimated. Although GAs have made significant progress, they still need to effectively balance exploring new areas of the solution space and refining existing solutions.

As far as we know, there has been little research on recognizing emotions based on gender or exploring how gender influences emotion recognition. Furthermore, there has not been any investigations into which features impact emotion recognition. Our study aims to fill this gap. In recent years, GAs have become widely known for their great potential in solving complex optimization problems. Therefore, we introduce a novel GA for SER, and the main contributions of this paper are summarized as follows:

1. Propose a novel speech gender–emotion recognition model.
2. Extract various features from speech for gender and emotion recognition.
3. Utilize a genetic algorithm for high-dimensional feature selection for fast emotion recognition, in which the algorithm is improved through feature evaluation, the selection of parents, crossover, and mutation.
4. Validate the performance of the proposed algorithm on four English datasets.

The structure of this paper is arranged as follows: Section 2 explains the related works on SER, and the proposed model is presented in Section 3. Section 4 contains the experimental results and discussions, while Section 5 provides the conclusions.

## 2. Related Works

Gender-based SER systems are designed to detect and analyze emotional states in spoken language, with a focus on distinguishing between male and female speakers. These systems aim to identify emotional signals, such as tone, pitch, intensity, and other acoustic features, and associate them with particular emotions.

Bisio et al. recognized individuals’ emotional states by registering audio signals, and their system had two functions: gender recognition (GR) and emotion recognition (ER) [27]. GR was implemented through a pitch frequency estimation approach, while ER used an SVM classifier based on correctly selected audio features. The performance analysis revealed that the emotion recognition system achieved high recognition rates and accurately identified emotional content. Bhattacharya et al. studied the influence of multimodal emotional features produced by facial expressions, voice, and text [28]. By analyzing a substantial dataset comprising 2176 manually annotated YouTube videos, they noticed that the performance of multimodal features consistently surpassed that of bimodal and unimodal features. This performance variation was caused by different emotional contexts, gender factors, and video durations. In particular, male speakers exhibited strong suitability for multimodal features in identifying most emotions. Zaman et al. presented a system for recognizing age, gender, and emotion from audio speech [29]. In this system, all audio files were converted into 20 statistical features and the transformed digital datasets were employed to develop various prediction models, such as artificial neural networks, CatBoost, XGBoost, AdaBoost, gradient boosting, KNN, random forest, decision tree, naive Bayes and support vector machine (SVM) models. CatBoost demonstrated the highest performance among all the prediction models.

Speaker recognition systems often experience a decline in performance in the presence of emotional or stressful conditions. Verma et al. examined the intonation and stress patterns of speech in Hindi (Indo-Aryan) and evaluated the impact of gender on the accuracy of SER [30]. This study suggested a system that recognized both gender and emotion through obtaining fundamental prosodic and spectral speech features, and then compared three classification algorithms. Experiments on the Hindi emotion corpus revealed that the SVM achieved 78% accuracy in recognizing speech emotions. Bandela et al. proposed a novel feature extraction method using the teager energy operator (TEO) to detect stress emotions. TEO was specifically designed to increase the energy of accented speech signals [31]. For gender-dependent and speaker-independent conditions, a KNN classifier was used for emotion classification in EMA (English), EMOVO (Italian), EMO-DB (German), and IITKGP (Telugu) databases. Rituerto et al. introduced a speaker recognition system designed for personalized wearable devices to address issues related to gender-based violence [32]. The objectives included measuring the impact of stress on speech and seeking ways to mitigate its effects on speaker recognition tasks. Given the scarcity of data resources for such scenarios, the system employed data augmentation techniques customized for this purpose.

These studies provided valuable insights into SER and related fields. However, they did not consider the variations in emotional features between male and female speakers. To address this, we employed a GA to efficiently recognize corresponding features and complete classification.

## 3. Materials and Methods

**Figure 1:**
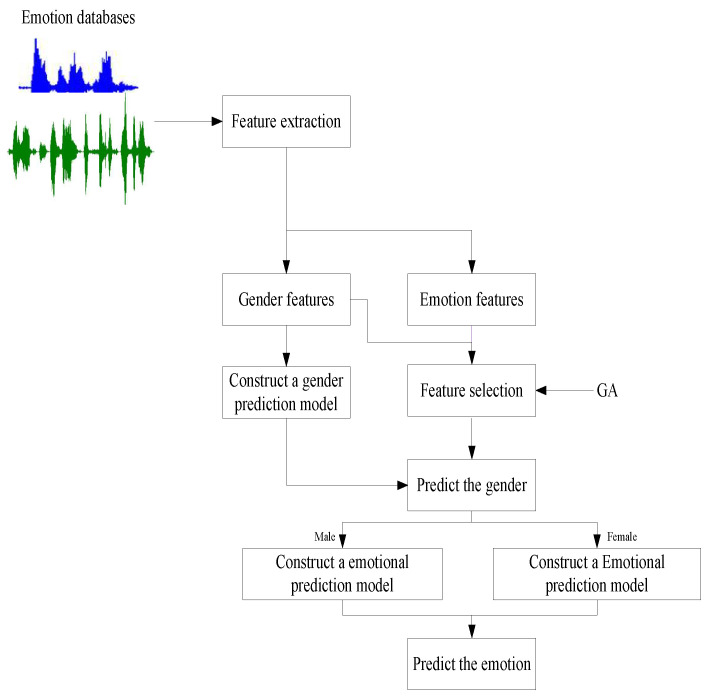
Flowchart of the proposed model.

Figure 1 depicts the proposed model, which includes emotional databases, feature extraction, and feature selection using an improved GA. This model establishes SER for males and females based on gender. When predicting emotions, it initially determines the gender of the voices, and subsequently employs the relevant emotion model based on that gender prediction.

### 3.1. Emotional Databases

In this study, we utilized four datasets: CREMA-D [3], EmergencyCalls [33], IEMOCAP-S1 [34], and RAVDESS [35], for gender-based emotion recognition. These databases are valuable for research in emotion recognition, speech analysis, and related fields. They provide a range of emotional expressions, and they are useful resources for developing and testing emotion recognition algorithms and models.

1 CREMA-D

CREMA-D is a dataset comprising 7442 original video clips performed by 91 actors. Among these actors, 48 are male, and 43 are female, with ages ranging from 20 to 74. The actors delivered a series of 12 sentences that expressed one of six distinct emotions: angry, disgust, fearful, happy, sad, and neutral. These emotions are depicted at four different intensity levels: low, medium, high, and unspecified.

2 EmergencyCalls

The 18 speakers were instructed to record their voices portraying four different emotions: angry, drunk, painful, and stressful. After labeling, audio files were cleaned to eliminate any background noise. Subsequently, the 338 recordings were trimmed to a uniform length of approximately 3 s each. Additionally, synthetic audio files were generated by adjusting the pitch of recordings from four selected speakers.

3 IEMOCAP-S1

The Interactive Emotional Dyadic Motion Capture (IEMOCAP) database is an acted, multimodal, and multispeaker database recently collected at the SAIL lab at USC. It contains approximately 12 h of data, including text, speech, video, and facial transcriptions. Actors perform both scripted and spontaneous dialogues, expressing a range of emotions, such as angry, happy, sad, and neutral, as well as dimensional labels such as activation, valence, and dominance. The IEMOCAP dataset is highly regarded for its detailed annotation of emotional expressions and multimodal nature. IEMOCAP-S1 is the S1 session of IEMOCAP, and it contains 1819 video clips.

4 RAVDESS

RAVDESS is a database that contains audiovisual recordings of individuals expressing a wide range of emotional states through speech and song. RAVDESS comprises 7356 audio and video clips, and each clip lasts approximately 3 to 5 s. The database is composed of 24 professional actors (12 male and 12 female). The RAVDESS database covers various emotions, including angry, happy, sad, surprised, fearful, and disgust.

The datasets contain an equal number of male and female speakers. CREMA-D, EmergencyCalls, and RAVDESS provide enough emotional samples to balance the numbers. The sample data in IEMOCAP-S1 is unevenly distributed, especially for fearful, other, and disgust emotions, which make up only 3% of the samples.

### 3.2. Feature Extraction

We extracted pitch and acoustic features from the databases introduced in Section 3.1. Pitch features were used to identify gender and the OpenSmile toolkit was employed to obtain acoustic features for emotion recognition. Table 1 describes their details, in which gender contains 11 features and emotion has 485 values.

### 3.3. Improved Genetic Algorithm

**Figure 2:**
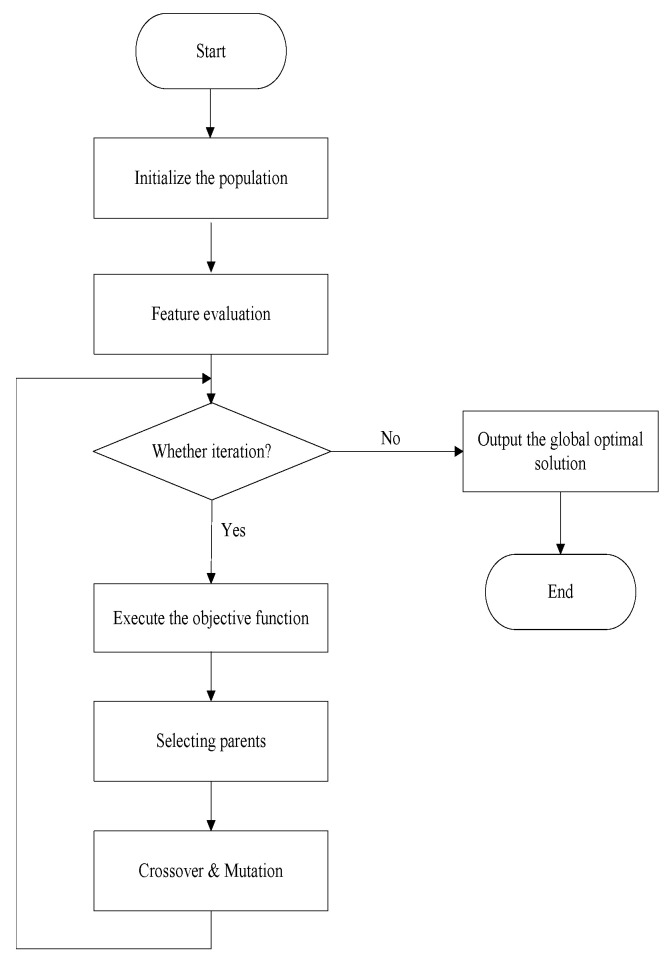
Flowchart of the IGA.

Since a large number of features were extracted from the speech signals, we employed a GA to perform feature selection, to reduce the dimensionality of features and improve the recognition accuracy. GAs have good a global search ability, and they are widely used to solve optimization problems. The classical GA performs well in the early stages. However, as the population approaches the global optimum, the diversity within the population gradually diminishes. GAs are prone to prematurity and may become stuck in local optimality too early. An improved GA (IGA), based on feature evaluation, the selection of parents, crossover, and mutation, was proposed, to increase the diversity of the population and balance global search and local convergence, as shown in Figure 2.

#### 3.3.1. Feature Evaluation

**Figure 3:**
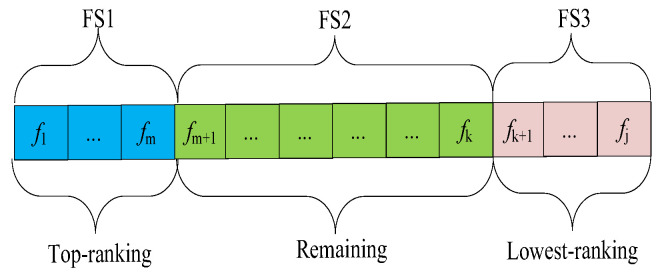
Feature space division.

Fisher score is a statistical measure used in feature selection to evaluate the discriminatory ability of features in distinguishing between different classes in a dataset [36,37]. The equation for Fisher score is given as follows:

(1) $$F=\frac{Between-ClassVariance}{Within-ClassVariance}$$

where Between−ClassVariance is the variance among the means of different classes, while Within−ClassVariance is the average variance within each class. A higher Fisher score indicates better discrimination between classes. In feature selection, features with higher Fisher scores are considered more relevant, and they are more likely to contribute significantly to the separation of classes. Based on the Fisher score of features, we divided the feature space into three subspaces: the top-ranking features (FS1) accounted for 10%; the lowest-ranking features (FS3) accounted for 10%; the remaining features (FS2) accounted for 80%, as shown in Figure 3.

#### 3.3.2. The Selection of Parents

The parents in a GA typically adopt the roulette wheel selection method based on their performance. The population will quickly converge if parents are generated near the optimal value. However, they cannot maintain their convergence speed and search ability. Additionally, as the algorithm searches, its ability to explore new solutions decreases due to the approximation of the optimal solution. When producing the next population, one should select excellent parents as much as possible, and then adjust the selection of parents according to offspring during the search process to expand the search scope. Algorithm 1 displays the new parent selection scheme.

**Algorithm 1:** The selection of parents

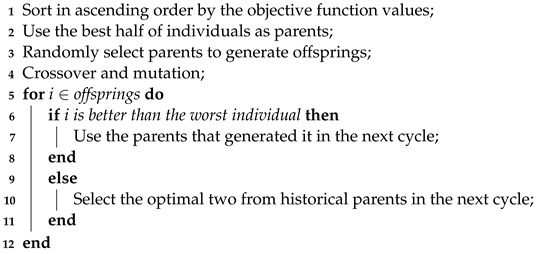



To enhance the convergence speed, we made a change to the algorithm so that only the top-performing half of parents participated in generating offspring during each cycle. As illustrated in line 5 of the algorithm, if the newly generated offspring are excellent, their parents will continue to be used in the next cycle. However, if they do not meet this criterion, the algorithm employs the previous excellent solutions that are not involved in the current cycle as parents.

#### 3.3.3. Crossover and Mutation

In high-dimensional feature selection, features from FS1 have a high probability of being selected in the mutation of offspring. Meanwhile, features from FS2 have a low probability of being selected. We proposed a new crossover and mutation method for gender differences. Due to the lack of knowledge about which features affect emotional recognition in males and females, their initial values remained the same to reduce bias. As the algorithm was executed, it gradually identified gender-specific features. The dimensions were divided into two parts: one half is dedicated to detecting male emotional features, while the other half focuses on identifying female emotional features. As a result, the dimensions of the population are twice the number of features in the datasets.

**Figure 4:**
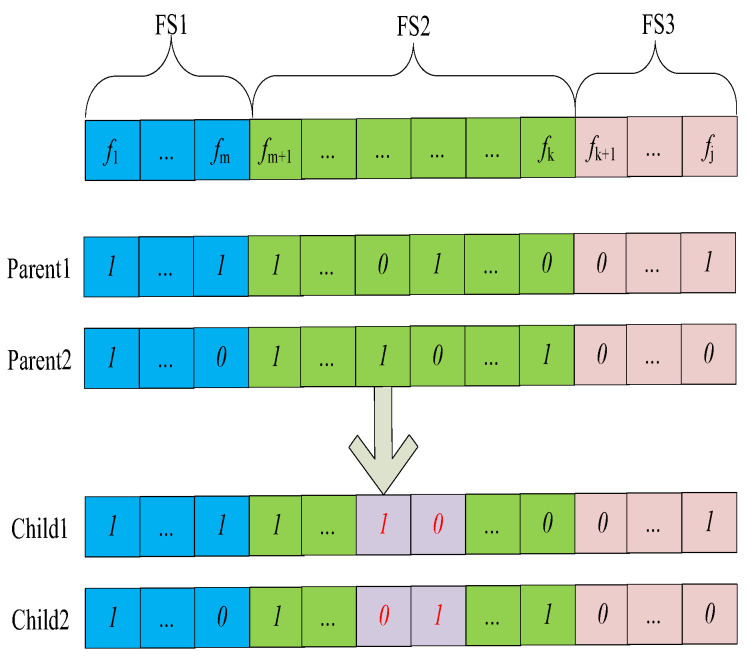
Crossover.

Crossover mimics the genetic recombination found in nature and aims to create new solutions by fusing the characteristics of promising parents. As depicted in Figure 4, features from FS1 are more likely to be selected, whereas features from FS3 are typically not chosen. Because their crossover does not significantly enhance genetic diversity, they are excluded from the crossover operation. The number of intersection points of FS2 is randomly generated within the range of 1 to 5.

Mutation introduces new genetic material into the population by randomly changing the values of several genes in the chromosomes, thereby maintaining genetic diversity. These changes enable the GA to explore areas of the solution space that would otherwise be inaccessible. During the mutation process, it is crucial to consider gender and feature differences. We developed a new mutation method that takes these factors into account, as illustrated in Algorithm 2.
**Algorithm 2:** Mutation
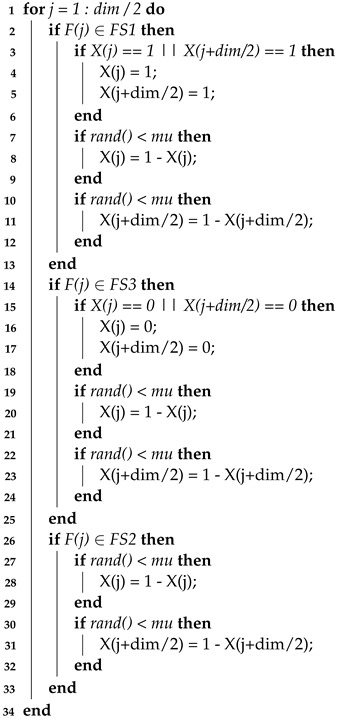


The features of FS1 and FS3 are ranked as the most important or least important. Lines 1–25 indicate that if either male or female features from FS1 and FS3 are selected/unselected, then the corresponding features for the other gender should also be selected/unselected. Additionally, the algorithm’s diversity is increased through mutation. Lines 26–33 utilize the GA’s mutation scheme to search for gender-differentiated features.

## 4. Experimental Results and Analysis

To assess the effectiveness of our proposed IGA, we compared its performance with the GA [38], BBO_PSO [39], and MA [40]. We used the default parameter settings for all algorithms, to ensure a fair comparison, and Table 2 presents their main parameters setting.

It is worth noting that BBO_PSO does not incorporate gender information, while GA and IGA employ a gender–emotion model as illustrated in Figure 1.

The population size of the algorithms was 20, and they were run 20 times. The maximum evaluations for IGA, GA, BBO_PSO, and MA were 2000. To evaluate the statistical significance of the experimental results, we employed the Wilcoxon rank sum test and the Friedman test. A significance level of 0.05 was chosen to examine if there were any noteworthy differences in the obtained results.

### 4.1. Objective Function

The primary metric of SER is classification accuracy. Therefore, we utilized this as the objective function in our experiments, as depicted in Equation (2). Additionally, we assessed the algorithms in terms of precision, recall, F1-score, the number of selected features, and the time taken for execution.

(2) $$accuracy=\frac{TP+TN}{TP+TN+FP+FN}$$

(3) $$precision=\frac{TP}{TP+FP}$$

(4) $$recall=\frac{TP}{TP+FN}$$

(5) $$F1-score=\frac{2*TP}{2*TP+FP+FN}$$

where TP stands for the number of instances that were actually positive and correctly predicted as positive. TN represents the number of instances that were truly negative and accurately predicted as negative. FP signifies the number of instances that were actually negative but incorrectly predicted as positive. FN indicates the number of instances that were truly positive but wrongly predicted as negative.

### 4.2. Experimental Analysis

We employed a SVM and 10-fold cross-validation to evaluate the performance of the SER algorithms.

Table 3 displays the average recognition accuracy of the algorithms. The improvement strategies of the IGA appeared to be effective, as it achieved a better classification accuracy than the GA on CREMA-D, EmergencyCalls, IEMOCAP-S1, and RAVDESS. The IGA outperformed the MA, BBO_PSO, and GA on EmergencyCalls, IEMOCAP-S1, and RAVDESS, and especially on EmergencyCalls, it exhibited significant superiority. The MA surpassed the compared algorithms only on CREMA-D. Compared to the two current emotion recognition algorithms, BBO_PSO and MA, the IGA improved the accuracy by 10% on EmergencyCalls, 3% on IEMOCAP-S1, and 2% on RAVDESS. The algorithms performed better on CREMA-D, EmergencyCalls, and RAVDESS than on IEMOCAP-S1. EmergencyCalls, IEMOCAP-S1, and RAVDESS have fewer emotions than IEMOCAP-S1, and they have a more uniform data distribution, which can facilitate constructing efficient SER models.

The results of the Friedman test demonstrated that the IGA achieved the best performance on three out of four datasets, while the IGA was inferior to the MA only on EmergencyCalls. BBO_PSO performed the worst. Furthermore, it is noteworthy that the IGA, MA, and GA utilized gender features to recognize speech emotions, which implies that gender information can improve the accuracy of SERs. The Wilcoxon rank sum test indicated that the MA, BBO_PSO, GA, and IGA performed well on 1, 0, 0, and 3 emotional datasets, respectively. The MA and IGA could not distinguish the experimental data in RAVDESS within a 5% confidence interval. Therefore, the IGA was more effective than the other algorithms.

**Figure 5:**
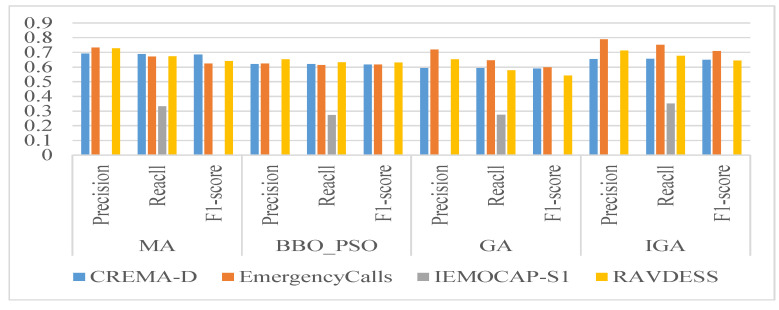
The precision, recall, and F1-score of the algorithms.

Figure 5 shows the precision, recall, and F1-score of the algorithms. Similarly to the observations in Table 3, the algorithms demonstrated superior performance on EmergencyCalls and RAVDESS when compared to CREMA-D. The algorithms identified fearful, other, and disgust emotions as other emotions, but they did not mistakenly classify other emotions as these specific emotions. Their values in precision and F1-score were NaN. BBO_PSO was inferior to the MA, GA, and IGA. These algorithms had better precision over recall, and they accurately recognized correct emotional samples.

Table 4 presents the number of selected features and running time of the algorithms. Even though the GA acquired more features than the MA and BBO_PSO, the IGA and GA managed to search twice as much feature space as the MA and BBO_PSO. Remarkably, the IGA used the lowest number of features for recognition. The IGA divided the feature space through Fisher score and avoided wasting searches on less important features. It was possible to find supplementary features (from FS2) that were suitable for classification through importance-based crossover and mutation. The number of extracted features from these datasets was equal, so the algorithms did not achieve a significant difference for the number of selected features. The execution time of the feature selection algorithms was mainly affected by their objective function. The SVMs’ time complexity ranged from O($N*D^{2}$) to O($N*D^{2}$), where *N* means the feature size and *D* implies the number of samples. These algorithms are more efficient when there are fewer features used for a given dataset. Consequently, the execution time of the IGA was shorter than that of the other algorithms. Additionally, it is important to highlight that CREMA-D, with its larger sample size, tended to have the longest execution time, while EmergencyCalls, with fewer samples, led to a shorter execution time for the algorithms.

Based on the above analysis, the IGA demonstrated outstanding performance in various evaluation metrics, and it is suitable for sentiment analysis of English speech.

### 4.3. Discussion

**Figure 6:**
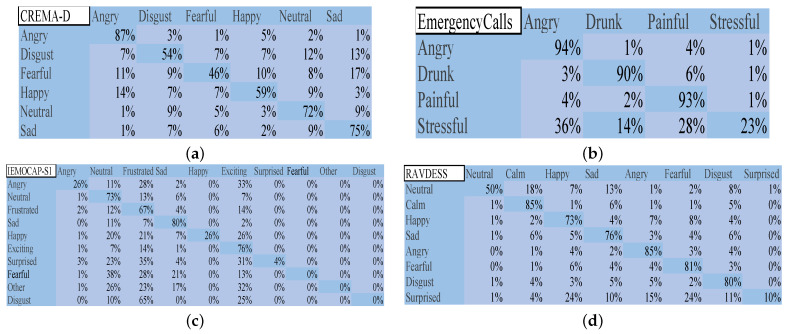
Confusion matrix of IGA. (**a**) CREMA-D; (**b**) EmergencyCalls; (**c**) IEMOCAP-S1; (**d**) RAVDESS.

Figure 6 displays the confusion matrix of IGA. On CREMA-D, the IGA faced challenges in distinguishing between neutral, sad, and disgust emotions. It struggled to classify fearful accurately, and the presence of the other five emotions could interfere with its recognition. On EmergencyCalls, the IGA performed exceptionally well in recognizing angry, drunk, and painful emotions, but these emotions also impacted its ability to judge stressful effectively. On IEMOCAP-S1, the neutral, frustrated, and exciting emotions negatively affected the IGA’s recognition of other emotions. The algorithm also tended to misclassify sad as fearful and other. On RAVDESS, sad influenced the classification of neutral, and emotions were confused with surprised, particularly for happy, sad, and fearful.

On CREMA-D, the GA, BBO_PSO, and MA showed similar results to the IGA. Identifying fearful was easier for the GA than the IGA, while BBO_PSO outperformed the IGA in recognizing happy emotions. The MA achieved a better accuracy than the IGA in recognizing disgust, fearful, happy, and neutral emotions. On EmergencyCalls, painful emotions significantly affected the MA’s ability to recognize stressful. The GA and BBO_PSO often misclassified stressful as angry and painful. On RAVDESS, the BBO_PSO performed exceptionally well in recognizing surprised, with an accuracy of 73.7%. On IEMOCAP-S1, the GA, BBO_PSO, and MA could not identify neutral, frustrated, and exciting emotions, while the BBO_PSO misclassified surprised as exciting.

The acoustic features of males and females are generally different, in the following ways:

(1) In CREMA-D, the second derivative of PCM, and the variances in logMelFreqBand[6] and logMelFreqBand_FDE[2] are the features with the most differences. Variance is an extremely influential statistical characteristic, and logMelFreqBand[0–7] stand out as the most important emotion features.
(2) In EmergencyCalls, the first derivative of logMelFreqBand_FDE[0] appears more frequently than the other features. Similarly to for CREMA-D, variance plays a significant role, and MFCC_FDE[0,2,3,5,6], logMelFreqBand[4,5,6], and logMelFreqBand_FDE[0,1,2,3,6] are identified as emotion features that exhibit gender differences.
(3) In RAVDESS, the first derivative of MFCC_FDE[12] and the quartile1 of PCM_FDE appear with a higher frequency. Mean and variance are the most important statistical characteristics, while MFCC[0,1,5,12], MFCC_FDE[0,10,11,12,14], PCM, jitterLocal, and jitterDDP are gender-difference features.
(4) In IEMOCAP-S1, the first derivative of logMelFreqBand_FDE[0], and the variances in logMelFreqBand[4,5] are features with distinct gender-related variations. Similarly to the previous datasets, variance maintains its importance. Notably, logMelFreqBand[0,1,2,7], MFCC[0,4,5], and their corresponding FDEs play influential roles in capturing gender-related emotional features.

## 5. Conclusions

Research has shown that people of different genders often express various emotions through their speech patterns, tones, and intonations. In our work, we built a speech emotion model based on gender to acquire a high accuracy, and this model involves extracting gender and emotion features, as well as feature selection. We employed higher-order statistics, spectral features, and temporal dynamics that might better capture the nuances between neutral, sad, and fearful emotions. The GA implements feature selection to improve the model’s ability to distinguish between similar emotions. In experiments on four English datasets, the accuracies of the proposed algorithm were 0.6565, 0.7509, 0.5859, and 0.6756 on the CREMA-D, EmergencyCalls, IEMOCAP-S1, and RAVDESS datasets, respectively. In terms of precision, recall, and F1-score, it was superior to the compared algorithms. Additionally, our research revealed that neutral, sad, and fearful emotions affect recognition accuracy, and the log mel frequency and MFCC are the main gender-differentiated emotional features. To improve the classification of challenging emotions such as stressful, fearful, and disgust, we could employ several strategies in our proposed model. We will use filter methods to comprehensively evaluate features and fine-tune the crossover and mutation parameters of the GA. We also intend to test different classifiers to evaluate their recognition performance. Additionally, data preprocessing will be used to enhance the quality and representativeness of the training set. This work could be extended by integrating facial and voice features from the same individual in various environments, which would improve recognition accuracy and the machine’s comprehension of human emotions.

## Figures and Tables

**Table 1:** Summary of gender and emotion features.

Category	Description	Features
Gender	Pitch	max, min, median, mean, variance and derivatives
Emotion	PCM loudness	max, min, median, mean, variance and derivatives
	MFCC [0–14]	max, min, median, mean, variance, derivatives, and their corresponding first-order delta coefficients (FDE) of smooth low-level descriptors
	log Mel Freq. Band [0–7]	skewness, kurtosis, max, min, median, mean, variance, derivatives, and FDE
	LSP Frequency [0–7]	max, min, median, mean, variance, derivatives, and FDE
	F0 by Sub-Harmonic Sum	lin. regression error Q/A-, max, min, median, mean, variance, derivatives, and FDE
	F0 Envelope quartile	quartile—1/2/3, max, min, median, mean, variance, derivatives, and FDE
	Voicing Probability	quartile range—2-1/3-2/3-1, max, min, median, mean, variance, derivatives, and FDE
	Jitter local	percentile 1/99, max, min, median, mean, variance, derivatives, and FDE
	Jitter DDP	percentile range 99-1, max, min, median, mean, variance, derivatives, and FDE
	Shimmer local	up-level time—75/90, max, min, median, mean, variance, derivatives, and FDE

**Table 2:** The main parameters setting.

Algorithms	Main Parameters
GA	pC = 1; mu = 0.02;
IGA	mu = 0.02;
BBO_PSO	pMutation = 0.1; KeepRate = 0.2;
MA	mu = 0.01; DANCE = 5; fl = 1

**Table 3:** The classification accuracy of the algorithms.

Datasets	MA	BBO_PSO	GA	IGA
CREMA-D	0.6890	0.6181	0.5923	0.6569
EmergencyCalls	0.6703	0.6077	0.6400	0.7471
IEMOCAP-S1	0.5671	0.4819	0.4928	0.6023
RAVDESS	0.6821	0.6532	0.5852	0.6838
>/≈/<	1/1/2	0/0/4	0/0/4	3/0/0
Rank	1.75	3.75	3.25	1.25
*p*-Value	0.0169			

**Table 4:** The number of selected features and running time of the algorithms.

Dataset	MA		BBO_PSO		GA		IGA	
	Length	Time	Length	Time	Length	Time	Length	Time
CREMA-D	317.4	40,543.1062	253.6	48,106.5402	461.1	20,755.5475	197.2	12,860.7447
EmergencyCalls	318.4	1433.4570	240.8	932.8905	482.65	706.4227	197.65	577.1138
IEMOCAP-S1	318.35	9930.3462	243.65	9985.9357	485.9	5127.9112	188.75	3476.7207
RAVDESS	319.95	8491.6366	245.45	6492.9197	492.3	4143.9636	193.5	2612.5911

## Data Availability

Data are available from the corresponding author on reasonable request, and this paper did not involve humans.

## References

[1] Bhushan B. (2023). Optimal Feature Learning for Speech Emotion Recognition—A DeepNet Approach. Proceedings of the 2023 International Conference on Data Science and Network Security (ICDSNS).

[2] Wani T.M., Gunawan T.S., Qadri S.A.A., Kartiwi M., Ambikairajah E. (2021). A comprehensive review of speech emotion recognition systems. IEEE Access.

[3] Donuk K. (2022). CREMA-D: Improving Accuracy with BPSO-Based Feature Selection for Emotion Recognition Using Speech. J. Soft Comput. Artif. Intell..

[4] Fahad M.S., Ranjan A., Yadav J., Deepak A. (2021). A survey of speech emotion recognition in natural environment. Digit. Signal Process..

[5] Akçay M.B., Oğuz K. (2020). Speech emotion recognition: Emotional models, databases, features, preprocessing methods, supporting modalities, and classifiers. Speech Commun..

[6] Issa D., Demirci M.F., Yazici A. (2020). Speech emotion recognition with deep convolutional neural networks. Biomed. Signal Process. Control.

[7] Hu G., Zhong J., Wang X., Wei G. (2022). Multi-strategy assisted chaotic coot-inspired optimization algorithm for medical feature selection: A cervical cancer behavior risk study. Comput. Biol. Med..

[8] Barrera-García J., Cisternas-Caneo F., Crawford B., Gómez Sánchez M., Soto R. (2023). Feature Selection Problem and Metaheuristics: A Systematic Literature Review about Its Formulation, Evaluation and Applications. Biomimetics.

[9] Hu P., Pan J.S., Chu S.C., Sun C. (2022). Multi-surrogate assisted binary particle swarm optimization algorithm and its application for feature selection. Appl. Soft Comput..

[10] Jia H., Rao H., Wen C., Mirjalili S. (2023). Crayfish optimization algorithm. Artif. Intell. Rev..

[11] Jia H., Peng X., Lang C. (2021). Remora optimization algorithm. Expert Syst. Appl..

[12] Hu G., Zhong J., Zhao C., Wei G., Chang C.T. (2023). LCAHA: A hybrid artificial hummingbird algorithm with multi-strategy for engineering applications. Comput. Methods Appl. Mech. Eng..

[13] Zhao W., Wang L., Zhang Z., Fan H., Zhang J., Mirjalili S., Khodadadi N., Cao Q. (2024). Electric eel foraging optimization: A new bio-inspired optimizer for engineering applications. Expert Syst. Appl..

[14] Wu D., Jia H., Abualigah L., Xing Z., Zheng R., Wang H., Altalhi M. (2022). Enhance teaching-learning-based optimization for tsallis-entropy-based feature selection classification approach. Processes.

[15] Lu J., Su X., Zhong J., Hu G. (2023). Multi-objective shape optimization of developable Bézier-like surfaces using non-dominated sorting genetic algorithm. Mech. Ind..

[16] Gao Y., Gao L., Liu Y., Wu M., Zhang Z. (2024). Assessment of water resources carrying capacity using chaotic particle swarm genetic algorithm. J. Am. Water Resour. Assoc..

[17] Pan J.S., Hu P., Snášel V., Chu S.C. (2022). A survey on binary metaheuristic algorithms and their engineering applications. Artif. Intell. Rev..

[18] Yue L., Hu P., Chu S.C., Pan J.S. (2023). Genetic Algorithm for High-Dimensional Emotion Recognition from Speech Signals. Electronics.

[19] Zhou J., Hua Z. (2022). A correlation guided genetic algorithm and its application to feature selection. Appl. Soft Comput..

[20] Song H., Wang J., Song L., Zhang H., Bei J., Ni J., Ye B. (2022). Improvement and application of hybrid real-coded genetic algorithm. Appl. Intell..

[21] Li J., Li L. (2020). A hybrid genetic algorithm based on information entropy and game theory. IEEE Access.

[22] Yan C., Li M.X., Liu W. (2019). Application of Improved Genetic Algorithm in Function Optimization. J. Inf. Sci. Eng..

[23] Harifi S., Mohamaddoust R. (2023). Zigzag mutation: A new mutation operator to improve the genetic algorithm. Multimed. Tools Appl..

[24] Dorea C.C., Guerra J.A., Morgado R., Pereira A.G. (2010). Multistage markov chain modeling of the genetic algorithm and convergence results. Numer. Funct. Anal. Optim..

[25] Li J.-H., Li M. (2013). An analysis on convergence and convergence rate estimate of elitist genetic algorithms in noisy environments. Optik.

[26] Peng Y., Luo X., Wei W. (2014). A new fuzzy adaptive simulated annealing genetic algorithm and its convergence analysis and convergence rate estimation. Int. J. Control Autom. Syst..

[27] Bisio I., Delfino A., Lavagetto F., Marchese M., Sciarrone A. (2013). Gender-driven emotion recognition through speech signals for ambient intelligence applications. IEEE Trans. Emerg. Top. Comput..

[28] Bhattacharya P., Gupta R.K., Yang Y. (2021). Exploring the contextual factors affecting multimodal emotion recognition in videos. IEEE Trans. Affect. Comput..

[29] Zaman S.R., Sadekeen D., Alfaz M.A., Shahriyar R. (2021). One source to detect them all: Gender, age, and emotion detection from voice. Proceedings of the 2021 IEEE 45th Annual Computers, Software, and Applications Conference (COMPSAC).

[30] Verma D., Mukhopadhyay D., Mark E. (2016). Role of gender influence in vocal Hindi conversations: A study on speech emotion recognition. Proceedings of the 2016 International Conference on Computing Communication Control and Automation (ICCUBEA).

[31] Bandela S.R., Siva Priyanka S., Sunil Kumar K., Vijay Bhaskar Reddy Y., Berhanu A.A. (2023). Stressed Speech Emotion Recognition Using Teager Energy and Spectral Feature Fusion with Feature Optimization. Comput. Intell. Neurosci..

[32] Rituerto-González E., Mínguez-Sánchez A., Gallardo-Antolín A., Peláez-Moreno C. (2019). Data augmentation for speaker identification under stress conditions to combat gender-based violence. Appl. Sci..

[33] Kaggle Speech Emotion Recognition for Emergency Calls. https://www.kaggle.com/datasets/anuvagoyal/speech-emotion-recognition-for-emergency-calls.

[34] Busso C., Bulut M., Lee C.C., Kazemzadeh A., Mower E., Kim S., Chang J.N., Lee S., Narayanan S.S. (2008). IEMOCAP: Interactive emotional dyadic motion capture database. Lang. Resour. Eval..

[35] Bajaj A., Jha A., Vashisth L., Tripathi K. (2022). Comparative Wavelet and MFCC Speech Emotion Recognition Experiments on the RAVDESS Dataset. Math. Stat. Eng. Appl..

[36] Mengash H.A., Alruwais N., Kouki F., Singla C., Abd Elhameed E.S., Mahmud A. (2023). Archimedes Optimization Algorithm-Based Feature Selection with Hybrid Deep-Learning-Based Churn Prediction in Telecom Industries. Biomimetics.

[37] Yao L., Yang J., Yuan P., Li G., Lu Y., Zhang T. (2023). Multi-Strategy Improved Sand Cat Swarm Optimization: Global Optimization and Feature Selection. Biomimetics.

[38] Sun L., Li Q., Fu S., Li P. (2022). Speech emotion recognition based on genetic algorithm–decision tree fusion of deep and acoustic features. ETRI J..

[39] Yogesh C., Hariharan M., Ngadiran R., Adom A.H., Yaacob S., Polat K. (2017). Hybrid BBO_PSO and higher order spectral features for emotion and stress recognition from natural speech. Appl. Soft Comput..

[40] Garain A., Ray B., Giampaolo F., Velasquez J.D., Singh P.K., Sarkar R. (2022). GRaNN: Feature selection with golden ratio-aided neural network for emotion, gender and speaker identification from voice signals. Neural Comput. Appl..

